# Neural stem cells fate under neuroinflammatory conditions and oxidative stress response

**DOI:** 10.3389/fncel.2025.1736865

**Published:** 2025-12-17

**Authors:** Filippo Torrisi, Simona Denaro, Jenny Ragonese, Simona D’Aprile, Agata Zappalà, Rosalba Parenti

**Affiliations:** 1 Department of Drug and Health Sciences, University of Catania, Catania, Italy; 2 Department of Biomedical and Biotechnological Sciences, University of Catania, Catania, Italy; 3 Department of Medicine and Surgery, University of Enna “Kore”, Enna, Italy

**Keywords:** redox homeostasis, differentiation, cytokines, NRF2, metabolic rewiring

## Abstract

Neural stem cells (NSCs) are defined by their self-renewal capacity and multipotent differentiation potential, making them essential for nervous system development and for the maintenance of adult brain homeostasis. Although confined to the subventricular zone and the subgranular zone of the hippocampus in adulthood, NSCs preserve a functional capacity for neurogenesis and tissue regeneration. This regenerative potential becomes particularly important in neuropathological conditions, where tissue damage is often accompanied by neuroinflammation and oxidative stress. Within this hostile microenvironment, NSCs have to cope with inflammatory mediators and reactive oxygen species that can affect their survival, proliferation, and cellular differentiation. NSCs also are actively modulated by diverse molecular pathways in response to stress conditions promoting stemness or stem cell exhaustion. Therefore, understanding the crosstalk between neuroinflammatory and oxidative stress in NSCs fate is crucial for elucidating the mechanisms of neurogenesis and homeostasis recovery and for designing therapeutic strategies.

## 1 Introduction

Neural stem cells (NSCs) are defined by their dual capacity for self-renewal and multipotent differentiation, maintaining a correct balance to ensure nervous system and long-term maintenance of neural homeostasis (Llorente et al., 2022). During embryonic and early postnatal stages, NSCs actively drive neurogenesis, contributing to the formation of cellular lineage for neural commitment and differentiation. However, in the adult brain, NSCs localize in at least two specialized canonical niches: the subgranular zone (SGZ) of the dentate gyrus in the hippocampus and the subventricular zone (SVZ) along the wall of the lateral ventricle (Llorente et al., 2022). Additional non-canonical or less active niches that may persist in adulthood include the hypothalamus, striatum, spinal cord, and cerebellum (Finkel et al., 2021; Batailler et al., 2016; Marichal et al., 2017). These stem cell populations retain the ability to generate neuronal or glial cells, thereby offering a unique reservoir of plasticity within the largely non-regenerative central nervous system (CNS) (Andreotti et al., 2019). This capacity, although modest, provides opportunities for compensating neural damage and restoring homeostasis, especially in pathological conditions.

Neuroinflammation can be considered a common hallmark and the most prevalent feature of CNS pathology across a wide spectrum of disorders, including neurodegenerative pathologies such as Alzheimer’s and Parkinson’s disease, ischemic and hemorrhagic stroke, traumatic brain and spinal cord injury, multiple and amyotrophic lateral sclerosis, spinal cord and brain tumors. The progression from acute to chronic inflammation is characterized by a complex and persistent immune response, where inflammatory mediators and prolonged reactive phenotypes of microglia and astrocytes give rise to oxidative stress within the neural microenvironment (Leszek et al., 2016; Russo et al., 2022). While inflammation is initially a protective mechanism, considered functionally important to remove harmful stimuli and initiate repair, its persistence often switches it into a driver of pathology. The transition from an acute, protective phase to a chronic inflammatory state contributes to progressive neurodegeneration, disruption of neural networks, and impairment of neurogenic potential (Kaniuk et al., 2025). Neuroinflammation is orchestrated by a dynamic interplay between resident glial cells, primarily microglia and astrocytes, and peripheral immune cells recruited to the CNS. Reactive microglia and infiltrating leukocytes release pro-inflammatory cytokines, chemokines, and cytotoxic mediators that shape the microenvironment of the neural niche (Mannino et al., 2022). Among the most detrimental consequences of this phenomenon, the production of reactive oxygen species (ROS), during the so-called respiratory burst, is of pivotal importance (Torres et al., 2006). While ROS are essential for host defense and redox signalling, their uncontrolled accumulation gives rise to oxidative stress, damaging cellular macromolecules, impairing mitochondrial function, and exacerbating tissue injury (Teleanu et al., 2022). NSCs respond to this oxidative and inflammatory environment by modulating their survival, proliferation, and lineage commitment, which may limit their regenerative potential. Nevertheless, NSCs are not passive targets of inflammation and oxidative stress. Evidence suggests that they actively interact with the surrounding microenvironment, sensing inflammatory cues and adapting their cellular functions (Hwang et al., 2021; Denaro et al., 2022). Depending on the nature, intensity, and chronicity of the inflammatory response, NSCs may undergo proliferation, shift their cell fate from stemness toward differentiation, or even contribute to modulating immune activity through paracrine signalling (Llorente et al., 2022). The correct balance of ROS signalling in neuroinflammatory conditions can be compromised, affecting NSCs fate and leading deterioration of the neurogenic niche (Hwang et al., 2021).

Understanding how NSCs respond to neuroinflammatory and oxidative stress conditions may provide insight into the fundamental mechanisms that regulate adult neurogenesis under both physiological and pathological states. The response of NSCs could be useful for improving regenerative strategies for a wide range of CNS disorders characterized by persistent inflammation and oxidative damage. Within this framework, the study of NSCs cell fate in neuroinflammatory conditions represents a key step toward defining their roles as potential agents of repair and functional restoration of the injured brain.

## 2 NSCs hallmarks of stemness and differentiation

### 2.1 The main molecular pathways

NSCs represent the basic reservoir from which the complexity of the nervous system originates. NSCs are the multipotent cells of the CNS capable of both self-renewal and differentiation into the main neural lineage, neurons, astrocytes, and oligodendrocytes (Llorente et al., 2022). During development, NSCs divide both symmetrically, to expand the stem cell pool, and asymmetrically, to generate daughter cells that progressively lose self-renewal and acquire lineage commitment. In this context, neural progenitor/precursor cells (NPCs) are considered more restricted descendants of NSCs. Despite their proliferative and differentiative capacities, they typically have limited self-renewal and narrower fate potential such as unipotent, bipotent, or restricted multipotent than NSCs (Nath et al., 2024; Homem et al., 2015). NSCs can be further classified into different functional states in response to inflammatory stimuli and activation of pathways such as TNF/MAPK/NFkB (Belenguer et al., 2021). In this review we will use the term NSCs to refer to the cell population with self-renewal and multipotency capabilities, and the term NPCs when referring to studies that indicate an effect on a specific population.

From a physiological point of view, the molecular control of the NSCs transition toward neurons or glial cells is orchestrated by a network of signalling pathways, transcriptional regulators and signals from the extracellular microenvironment (Navarro Quiroz et al., 2018). NSCs fate depends on inter, intra and extra cellular communications mediated by molecules acting as inducers of cellular development (Wen et al., 2009).

The connection between NSCs and inflammation arises from the dual role of cytokines, which, beyond their immune functions, are key regulators of neural embryogenesis. This is the case of bone morphogenetic protein (BMP), a member of the TGF-β family (TGF-β) which belongs to a signalling pathway that regulates embryogenesis, including the development of the neuroepithelial region, where neurons and epidermal cells are generated under their appropriate antagonism and synergistic effects (Meyers and Kessler, 2017). In the SVZ, NSCs generate neuroblasts that migrate to the olfactory bulb where under specific signals they differentiate into GABAergic and dopaminergic interneurons (Whitman and Greer, 2009). A network of molecular pathways and transcription factors work in a coordinated manner to preserve the stem cell state and to guide cells toward differentiation at the appropriate time. The fate of NSCs lies in the activation of specific molecular pathways that promote differentiation and modulate stem cell and self-renewal capacity. Therefore, NSCs fate lies in the activation of specific molecular pathways that promote differentiation and modulate stem cell and self-renewal capacity (Ahmed et al., 2009). The outcome of most inductive events is a change in DNA transcription, leading to the activation or repression of specific genes.

Stemness and differentiation are governed by key regulatory networks that integrate intercellular communication with chemical signalling pathways to preserve cellular integrity and identity. β-catenin is an anchoring protein for cell adherens junctions that attaches cadherins to actin filaments, acting also as a signalling molecule in one of the main molecular pathways implicated in neuronal development, called Wnt/β-catenin (Huang et al., 2015). In the canonical signalling, Wnt stabilizes β-catenin, a subunit of the cadherin protein complex, causing a blockage of the destruction complexes and thus allowing transcription factors T-cell factor/lymphoid enhancer factor (TCF/LEF) (Xue et al., 2025). Activation of transcription factors dislodges Groucho/TLE Co-repressor transcriptional repression complexes from target genes promoting the gene expression of specific marker of cell differentiation, tissue homeostasis and development. Wnt/β-catenin signalling upregulates proneural basic helix–loop–helix (bHLH) transcription factors such as NGN2 and NeuroD1, which promote neuronal differentiation (Lee et al., 2022). In NSCs, the repression of NeuroD1 by SRY sex-determining region-box 2 (SOX2) is overcome by Wnt signaling, which promote neuronal differentiation while concomitantly reducing their differentiation into oligodendrocytes and astrocytes (Kuwabara et al., 2009). Additionally, Wnt target gene is *CYCLIN D1* that is involved in asymmetrical proliferation of type 2a and 2b cells that differentiate into neuroblasts and direct their differentiation toward the formation of mature neurons with the intervention of NGN2, PROX1 and NeuroD1 (Arredondo et al., 2020). Wnt modulation or moderate activation does not force differentiation but supports NSCs proliferation and maintenance (He et al., 2018). In this context, the stem cell pool remains stable and dynamic, capable of both symmetric and asymmetric divisions.

Notch signalling is another critical molecular pathway that contributes to the maintenance of symmetric divisions during neurodevelopment to prevent premature differentiation, while in adults it primarily contributes to the quiescence of NSCs (Lampada and Taylor, 2023). Notch signalling depends on ligands binding, which triggers the cleavage of its intracellular domain NICD that translocate to the nucleus and regulates target genes trans by acting as a co-activator and co-repressor (Fabrizio et al., 2024). Notch can be considered a master signalling receptor in the process of lateral inhibition that prevents surrounding cells from differentiating in the same way and depends on a contact-dependent signalling mechanism activated by a Delta signalling protein. When an undifferentiated cell binds to Delta, Notch mediates the signal to stop differentiation (Bocci et al., 2020). In the Notch signalling pathway, basic helix–loop–helix (bHLH) transcriptional repressors such as Hairy and Enhancer of split homologs (HES) and HES-related type (HEY) form a heterodimer with strong repressive activity, suppressing the expression of pro-neural genes, thereby sustaining self-renewal (Sanalkumar et al., 2010). Therefore, Notch signalling is active and, through HES/HEY effectors, induces proneural genes repression, preventing premature differentiation (Ware et al., 2014). Conversely, loss of Notch activity results in upregulation of transcription factors such as Ascl1/Mash1, Neurog2, and NeuroD driving neuronal commitment. Ascl1 can bind and open up chromatin, facilitating the activation of the entire neurogenic program (Raposo et al., 2015). Upregulation of Neurog2 and NeuroD by bHLH transcription factors contribute to neuronal development by prompting progenitor cells to leave the cell cycle, promoting differentiation and maturation of both neuronal and glial cells (Matsushita et al., 2017).

A third molecular pathway associated with NSCs fate is Sonic Hegdehog-Gli (Shh-Gli). Shh is considered a morphogen capable of directing embryogenesis processes as a signal molecule (Vicario et al., 2019). The molecular mechanism of Shh is similar to Wnt since it is also a secreted signalling protein that acts as a local mediator and morphogen, activating proteins that regulate genes through transcriptional activation or repression (Torrisi et al., 2021). More specifically, transmembrane proteins such as Smoothened (Smo) and Patched are present downstream of Shh production. In the absence of a signal, Patched (Ptch1) keeps Smo inactive, while if Shh is active, it promotes the degradation of Ptch1, freeing SMO to translocate to the cell surface, transmitting a downstream signal that ends with the recruitment of a GLI protein that promotes the transcription of specific genes (Carballo et al., 2018). Shh has been mainly associated with the proliferative capacity of NSCs in both early and late embryonic stages (Takanaga et al., 2009). It maintains NSCs proliferation and expansion in early development, especially in the ventral forebrain and spinal cord, while gradual downregulation of Shh activity allows cells to shift toward mature neurons (Komada, 2012).

### 2.2 Key transcription factors and biomarkers

At the cellular level, it is important to note that the expression of Musashi-1, Notch1, and Stage-Specific Embryonic Antigen-1 (SSEA1) drive the differentiation of embryonic progenitor nervous cells into neuroepithelial cells, which subsequently give rise to radial glial cells. The latter are the progenitor cells of NSCs that can differentiate to oligodendrocytes, astrocytes, and neurons (Kriegstein and Alvarez-Buylla, 2009). Musashi-1 acts as an RNA binding protein, determining the repression of NUMB which is an inhibitor of Notch. In this way, activation of Notch signalling allows the maintenance of NSCs self-renewal (Okano et al., 2005). As NSCs begin to embark on the commitment pathway and become NPCs, the signalling pathways are reprogrammed. Notch signalling progressively declines, unleashing the action of pro-neural factors such as Ascl1 and Neurog2, which drive cells toward differentiation programs (Sueda and Kageyama, 2020). Shh signalling also declines, attenuating the constant proliferation and allowing cells to assume the state of amplifying progenitors, still proliferating but no longer indefinite (Komada, 2012). During this phase, Wnt signalling has a transient peak: the intense activation favors the numerical expansion of NSCs and, if sustained, directly initiates the neuronal program through TCF/LEF-mediated transcription. The presence of SSEA-1, an antigenic marker indicating a state of stemness or non-differentiation. Although known for its presence on the cell surface, proteomic studies have revealed that SSEA-1 in NSCs is transported by lysosomal membrane proteins such as Lysosome-Associated Membrane Protein 1 (LAMP-1) (Yagi et al., 2010). The expression of SSEA-1 bound to LAMP-1 disappears in mature cells as a result of stem cell differentiation. This suggests that the expression of SSEA-1-positive LAMP-1 could be a useful indicator of NSCs stemness and their ability to maintain the plasticity (Yagi et al., 2010).

The transition from neuroepithelial cells to NSCs leads to the rise of new biomarkers, such as Nestin and SOX2 which are maintained in radial glia. Nestin is an intermediate filament protein, implicated in the processes of self-renewal and NSCs survival (Park et al., 2010). The role of SOX2 is linked to the correct differentiation of NSCs from neuroepithelial cells to radial glial cells, which are then guided for maturation. SOX2 enhances Shh signalling thanks to GLI2, a downstream target of Shh. A similar feedback loop is observed with Epidermal Growth Factor Receptor (EGFR), whose expression is increased by SOX2, thus fostering EGFR signalling, which in turn increases SOX2 expression (Shimozaki, 2014). SOX2 is particularly critical for maintaining embryonic stem cells multipotency by directly repressing differentiation programs, cooperating with Octamer Transcription Factor 4 (OCT4) to activate genes for self-renewal, while simultaneously repressing developmental genes involved in lineage commitment (Sarkar and Hochedlinger, 2013). When expression of SOX2 is reduced, proneural factors like Ascl1 and Neurog2 dominate, establishing transcriptional programs that limit proliferation and direct lineage choice (Han et al., 2021).

As radial glial cells differentiate, new lineage-specific markers emerge: some are retained in astrocytic lineage, whereas others are lost with the acquisition of markers for oligodendrocytes and intermediate neuronal progenitors. Notch signalling decreases, allowing the full expression of proneural genes and the realization of terminal differentiation. Shh signalling can participate in the formation of oligodendrocytes, especially in ventral areas. However, in gliogenesis, a reduction in Wnt signalling in combination with STAT3 and NFIA favors astroglia commitment (Ma et al., 2024), while factors such as OLIG2 and NKX2.2 guide the oligodendroglial lineage (Talbott et al., 2005). Additional markers are: astrocyte-specific glutamate transporter (GLAST), that is a protein that marks NSCs as a type of astrocyte-like stem cell in the adult brain, contributing to adult neurogenesis (Llorens-Bobadilla et al., 2015); brain lipid-binding protein (BLBP), also known as FABP7, is a marker for a subpopulation of activated NSCs in the SVZ that is associated with mitotic activity, neurogenesis, and the generation of new brain cells, particularly in response to injury or aging, and it is also expressed by progenitor cells in the fetal and postnatal human brain (Giachino et al., 2014). BLBP identifies a specific subpopulation of NSCs that are actively dividing and have a high potential for generating neurons (Giachino et al., 2014). BLBP(+) NSCs express EGFR, which promotes their proliferation in response to EGF, making them a major clonogenic population in the SVZ (Giachino et al., 2014). At this stage, BLBP(+) NSCs remain proliferative but are lineage-restricted. As differentiation proceeds, cells exit the cell cycle and acquire a defined, post-mitotic identity.

**Figure 1:**
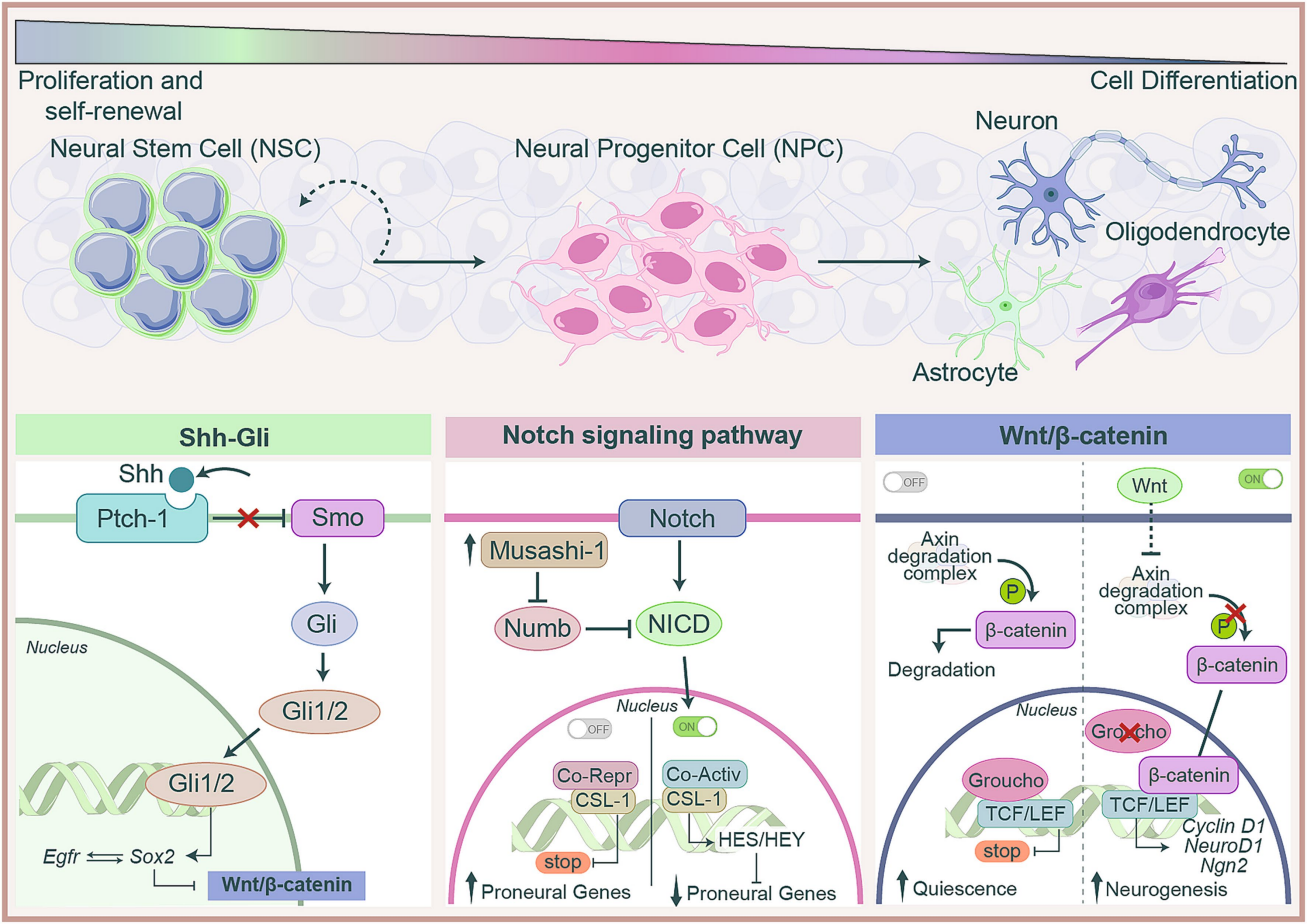
Molecular pathways regulating NSCs fate. NSCs gradually lose stemness and acquire specialized neuronal or glia identities through tightly regulated mechanisms. This transition depends on the integration of several signalling pathways that cooperate to balance self-renewal, proliferation and differentiation. In the absence of Sonic Hedgehog (Shh), Patched-1 (Ptch1) inhibits Smoothened (Smo), preventing activation of Gli transcription factors. When Shh binds to Ptch1, Smo allows the activation of Gli1/2, which translocate into the nucleus and promotes the transcription of genes involved in NSCs proliferation and stemness. Binding of Notch ligands triggers the release of the Notch intracellular domain (NICD), which enters the nucleus and associates with CSL-1 transcription factors to activate target genes such as HES and HEY, repressing proneural genes and maintaining stemness. Musashi-1, an RNA-binding protein localized in the cytoplasm, inhibits Numb, a negative regulator of Notch signalling, thereby reinforcing Notch activation ad preserving the undifferentiated state of NSCs. In the absence of Wnt, β-catenin is phosphorylated by the Axin complex and targeted for degradation, keeping TCF/LEF-bound target genes repressed by Groucho co-repressors, thus maintaining a quiescence state of NSCs. Upon Wnt signalling, the degradation complex is inhibited, leading to β-catenin stabilization and nuclear translocation. β-catenin associates with TCF/LEF to activate genes involved in neurogenesis. Altogheter, these pathways form and interconnected regulatory network that orchestrates NSC fate, finely regulating the progression from stemness to specialized neural lineages.

Summarizing the main key points of the markers and pathways that regulate NSCs fate, molecular regulations include the maintenance of self-renewal, expansion, and the control of genes that favor or hinder differentiation. Among the self-renewal programs that maintain stemness, Notch represents a dominant pathway, where the NICD/CSL/MAML complex induces HES1/2 to repress proneural genes such as Ascl1 and Neurog2 (Pierfelice et al., 2011). In this phase, the presence of Sox2 ensures stemness together with the Wnt/β-catenin pathway, and Shh participates by influencing gene transcription in favor of genes that promote self-renewal (Pfannkuche et al., 2009; Parameswaran et al., 2014). Thus, the expansion of NSCs are ensured via multiple signalling pathways. GLI-1, activated downstream of the Shh pathway, promotes proliferation, while ID2, regulated by BMP/Wnt pathway and cooperating with Notch, contribute to maintain the proliferative state by blocking differentiation (Uribe et al., 2012). Signalling pathways and transcription factors are tightly interconnected, coordinating the processes of proliferation, self-renewal, differentiation, and repression of lineage-specific genes. Although many transcription factors act in a lineage-specific manner, the extensive cross-talk among signalling pathways creates a highly integrated regulatory network that needs a more in depth investigation (Figure 1).

## 3 Pathophysiology of NSCs under neuroinflammatory conditions

NSCs respond to cytokines and chemokines produced by CNS-resident cell populations, such as microglia and astrocytes, as well as to circulating and niche-derived factors from both the local and systemic environments (Willis et al., 2022). NSCs engage in dynamic interactions with surrounding cells, which enhances the complexity of their function in neuroinflammatory conditions. CNS resident immune cells, such as microglia, play a major role and can act before events involving peripheral immune cells. The innate immune component of neuroinflammatory processes is primarily mediated by microglia, the resident macrophages of the CNS parenchyma, which release ROS, cytokines, and chemokines to attract peripheral immune cells or act as antigen-presenting cells. In addition to microglia, astrocytes play a central role in CNS inflammation through their diverse reactive phenotypes and functions, becoming reactive in response to trauma, infection, or neurodegenerative conditions (Sofroniew, 2020; Denaro et al., 2024). Reactive astrocytes can adopt neurotoxic or neuroprotective phenotypes, and key pathways include JAK/STAT3, NFkB and MAPK, regulating production of inflammatory or protective molecules (Giovannoni and Quintana, 2020). As for TGF-β and BMP, NFkB proteins may represent a direct link between inflammation and neurogenesis since they are at the center of many stress, inflammatory, and immune responses but also play a key role in cellular development (Guo et al., 2024). Despite the most recent associations between NfkB and stemness have mostly been related to cancer stem cells (Mao et al., 2025), cytokines can activate NFkB by phosphorylating and ubiquitinating regulatory proteins, allowing their translocation into the nucleus for the transcription of key genes in NSCs proliferation, such as *CYCLIN D1* (Widera et al., 2008).

Covacu et al. reported a range of inflammatory cytokines and chemokines and their corresponding effects on NSCs, identifying factors that can either promote or inhibit proliferation, neurogenesis, and migration under neuroinflammatory conditions. These mediators can be broadly classified into: (i) Pro-inflammatory cytokines including interferon-*γ* (IFN-γ), interferon-*α* (IFN-α), tumor necrosis factor (TNF), interleukin-1β (IL-1β), interleukin-6 (IL-6), and interleukin-15 (IL-15); (ii) Anti-inflammatory cytokines, such as interleukin-4 (IL-4) and interleukin-10 (IL-10); Other inflammatory mediators including Leukemia Inhibitory Factor (LIF) and nitric oxide (NO), which can exert dual and context-dependent effects on NSCs fate (Covacu and Brundin, 2017).

### 3.1 Pro-inflammatory cytokine signalling and NSCs regulation

Pro-inflammatory cytokines and inflammatory mediators such as IL-6, IL-1β, TNF, and NO, released during neuroinflammation, are implicated in the regulation of NSCs proliferation and neuronal differentiation (Russo et al., 2011). Notably, IL-6, when combined with its soluble receptor (sIL-6R) to form the fusion complex Hyper-IL-6 (H-IL-6), promotes NSCs differentiation into neurons via the MAPK/CREB signalling pathway, giving rise to glutamatergic, GABAergic, and dopaminergic neuronal subtypes. In contrast, IL-6 alone is insufficient to induce differentiation, whereas the IL-6/sIL-6R complex (H-IL-6) activates gp130-dependent signalling, thereby triggering neuronal commitment (Islam et al., 2009). Chronic neuroinflammation contributes to neurodegeneration, resulting in cognitive impairment and reduced neuroprotective and regenerative activity of NSCs. For instance, conventional and innovative radiation therapy induce a remarkable phenotype reshaping, which may also negatively affect neurogenesis (Monje et al., 2002; Alberghina et al., 2024; Pisciotta et al., 2020; Torrisi et al., 2025). Moreover, adaptive immune cytokines, responding to reactive states induced by innate immunity signals, drive IFN-*γ* production that promotes neurogenesis and oligodendrogenesis (Baron et al., 2008; Pereira et al., 2015). In addition, in a recovery model of West Nile virus encephalitis, persistent astrocyte inflammasome activation leads to excessive IL-1β production in NSCs, which limits synaptic repair within the hippocampal CA3 region, further impairing neuronal activity and cognitive recovery (Soung et al., 2022). A similar study evaluating persistent inflammation in the brain reported a significant alteration of olfactory neurogenesis associated with elevated expression of IFN*γ* and IL-6 in a mouse model of neuron-restricted measles virus MeV infection (Chandwani et al., 2023). IL-1β induces neuronal differentiation in cortical neural precursor cells through noncanonical Wnt5a/RhoA/ROCK/JNK pathway, promoting neurite outgrowth (Park et al., 2018); a similar increase of neurite growth was reported by Boato et al. after co-administration of IL-1β and neurotrophin-3 (Boato et al., 2011). It has been shown that JNK signalling is crucial for neural cell maturation and differentiation, regulating NSCs proliferation and differentiation during development, neuronal migration, and the formation of neural structures like axons and dendrites (Castro-Torres et al., 2024); activation of the JAK2/STAT4/STAT5 signalling pathway upregulates IFN-*γ* and IL-10 expression in CD4^+^ T cells cocultured with hippocampal NSCs from a mouse model of Alzheimer’s disease, thereby promoting their differentiation (Wu et al., 2022); conversely, IL-10 has been reported in maintaining NPCs in an undifferentiated state upregulating neural gene markers of progenitors cells and downregulating neuronal gene expression (Perez-Asensio et al., 2013); thus, the effects on NSCs fate depend on the specific cellular context and cell types involved, hence, elucidating the direction and interplay of the underlying signalling pathways is crucial. Other studies pointed out an essential role of IFN-γ in maintaining homeostasis in traumatic phenomena such as spinal cord injury (Roselli et al., 2018). Indeed, it has been reported that an accumulation of CD8 + T cells suppress NSCs proliferation and the differentiation into astrocytes through the IFN-γ-STAT1 pathway (Lima, 2023). Spinal cord injury recovery effects have also been observed in relation to SAM and SH3 domain-containing protein 1 (SASH1) whose inhibition determines a decreased IFN-γ and an increased brain-derived neurotrophic factor (BDNF) release (Liu et al., 2023). Moreover, JAK/STAT and Wnt/β-catenin were found linked through STAT1 recruitment into the promoter of β-catenin under the effect of IFN-γ, promoting NSCs proliferation, self-renewal, and differentiation; co-delivery of IFN-γ and NSCs facilitated their ability of neurological repair after ischemic stroke (Yuan et al., 2020; Zhang et al., 2018). These findings highlight a complex interplay between inflammatory cytokines, Wnt signalling, and JAK/STAT pathways in regulating NSCs fate and neuronal maturation, with IFN-γ exerting dual, context-dependent effects supporting homeostatic regulation under pathophysiological conditions. Notably, as chronic inflammation is also a hallmark of cancer, similar mechanisms may operate within the tumor microenvironment. In glioma, exposure of tumor cells to NSCs-conditioned medium results in downregulation of the Wnt/β-catenin pathway, where secreted IFN-*α* acts in a paracrine manner to inhibit cell proliferation, migration, and invasion (Yin et al., 2023). Together, these observations suggest that inflammatory cytokine signalling can act as a common axis linking neural dysfunction and tumor progression through context-dependent modulation of Wnt and JAK/STAT pathways. Beyond their well-established association with immunosuppressive mechanisms, gliomas also exhibit tumorigenic processes that may arise from dysregulation of neuroinflammatory programs governing NSCs. Key signalling pathways and cytokines active in glioma stem cells such as Wnt/β-catenin, which regulates stemness, and TGF-β, which promotes an immunosuppressive state have been linked to ERK phosphorylation, a critical mediator controlling the differentiation balance between proneural and mesenchymal phenotypes (Santoni et al., 2021; D'Aprile et al., 2024). The connection between NSCs and gliomas revealed that paracrine communication between these two cell populations is mediated by IFN-α, which inactivates the Wnt/β-catenin signalling pathway (Yin et al., 2023). Such evidence suggests that NSCs are not just passive receivers but can be a source of IFN-α. The negative regulatory effect on neurogenesis in adult brain has been reported by Zheng et al., showing that chronic IFN-α exposure leads to fewer proliferating NSCs and a reduced number of newborn neurons in hippocampus (Zheng et al., 2014).

Among the inflammatory mediators influencing NSCs dynamics, TNF plays a particularly multifaceted role in balancing activation and quiescence within the NSCs pool. Specifically, TNF is involved in a signalling pathway that regulates the shift between active and dormant states via a transient and reversible quiescent phase, called shallow quiescence, characterized by NSCs with low levels of EGF receptor (EGFR) and glutamate/aspartate transporter (GLAST). These shallow quiescence cells can either be primed for activation via TNF receptor 2 (TNFR2) or driven toward deeper quiescence through TNF receptor 1 (TNFR1) mediated by MAPK p38 phosphorylation and NFκB-dependent transcriptional activity (Belenguer et al., 2021). The NSCs quiescence associated with the TNF-TNFR2 pathway could rationalize immunomodulatory properties of NSCs for the immunosuppressive mechanisms that can be translated into other pathological conditions of neuroinflammation (Shamdani et al., 2020). The involvement of NSCs and TNF levels in neuroinflammatory conditions has also been associated with neurological recovery processes. TNF inhibition supports the neurological recovery processes mediated by polylactic acid and graphene oxide composites in spinal cord injury models shifting the immune balance toward a pro-regenerative macrophage phenotype and decreasing pro-inflammatory populations (Liu et al., 2025). Recovery from spinal cord injury associated with a decrease in TNF levels has been observed after transplantation of preconditioned NSCs under hypoxic conditions, showing good cell motility capabilities mediated by the activation of cell–cell adhesion, cell proliferation, and cell cycle (Fan et al., 2022). The role of TNF in inflammatory processes following neurological injury is crucial, but at the same time potentially harmful if its production is not modulated. In this context, NSCs-derived conditioned medium has been shown to significantly reduce the infiltration of pro-inflammatory mediators, including TNF, and limit the accumulation of CD68^+^ inflammatory macrophages in the sciatic nerve (Chen et al., 2020). This modulation of the immune environment translates into enhanced structural protection, as demonstrated by NSCs transplantation in a sciatic nerve injury model, which promoted myelin sheath recovery that was accompanied by reduced levels of IL-1β and TNF (Zhang et al., 2021). In experimental models of focal cerebral ischemia induced by middle cerebral artery occlusion, NSCs transplantation has further shown a neuroprotective effect. This is achieved through activation of the GDNF/PI3K/AKT axis, resulting in improved neurological function and a significant reduction in key inflammatory mediators such as IL-6, IL-8, and TNF (Xu et al., 2023). Inhibition of TNF–induced necroptosis via the RIPK1/MLKL pathway has been shown to enhance the survival of transplanted NSCs and promote sustained functional recovery over time (Tong et al., 2023). In summary, the synergistic action of NSCs leads to reduced inflammatory burden mediated by cytokines and promotes cell survival and regeneration pathways. This dual effect opens significant prospects for the use of NSCs as a biological therapy in post-traumatic neurological recovery processes.

### 3.2 Anti-inflammatory cytokine signalling and NSCs regulation

The immunomodulatory potential of anti-inflammatory cytokines in relation to NSCs has been investigated in many studies to observe the recovery of physiological conditions after neuroinflammation (Dooley et al., 2014). IL-4 has been shown to rescue the loss of NSCs plasticity, through reduction of KAT2, the enzyme responsible for kynurenic acid synthesis, which has been found upregulated after Amyloid-β42 (Aβ42) exposure (Papadimitriou et al., 2018). Such effects can be related to the role of IL-4 as the key molecule through which NSCs exert their effects on macrophages, by suppressing their inflammatory phenotype as reported in a study of *in vitro* co-culture (Ji et al., 2020). The beneficial immunosuppressive function of IL-10 and IL-4 for the migration of NSCs to sites of inflammation in association with an increase of chemokine receptor expression was reported in experimental autoimmune encephalomyelitis model (Guan et al., 2008). IL-4 and IL-10 stimulate microglia promoting phagocytic activity by the up-regulation of triggering receptor expressed on myeloid cells-2 (TREM2) (Yi et al., 2020). Similarly, enhancing the immunomodulatory capacity NSCs via TGFβ1 transduction significantly increased regulatory T cell development and shifted macrophages and microglia toward a pro-regenerative phenotype (Xie et al., 2018). Consistently, IL-10–engineered clinical-grade mesenchymal stromal cells showed marked therapeutic benefits in a spinal cord injury model, where they reduced lesion volume, improved functional recovery, and promoted axon regeneration, neuronal survival and differentiation modulatory effects on macrophages inflammatory phenotypes (Gao et al., 2022).

**Figure 2:**
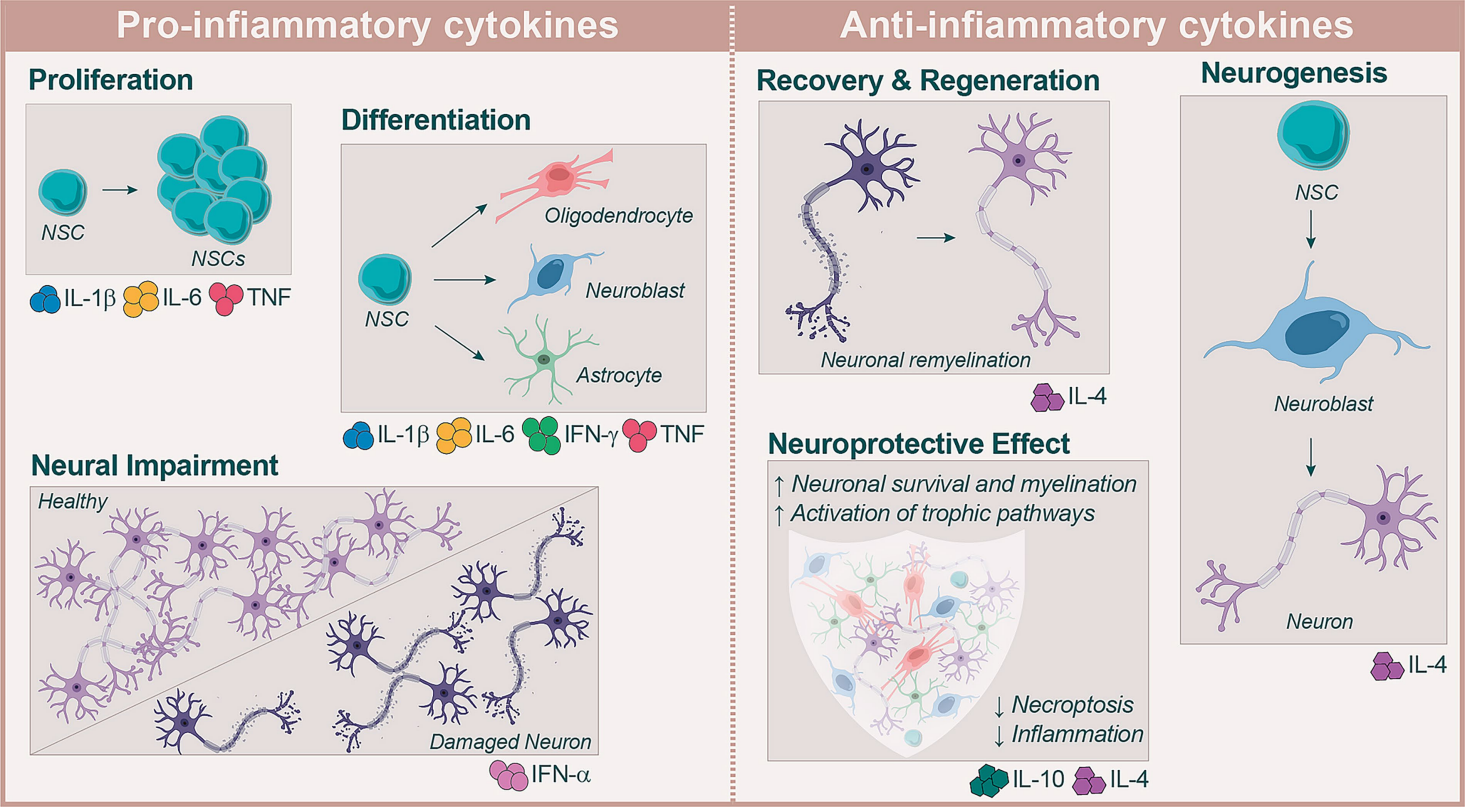
Microglia and reactive astrocytes in the CNS, such as microglia and astrocytes, secrete pro-inflammatory cytokines, which are depicted in the section on the left. These astrocytes adopt neurotoxic or neuroprotective phenotypes, regulated by key pathways such as JAK/STAT3, NF-κB and MAPK. The actions of these mediators on NSCs are multifaceted and often dual in nature: while they can promote proliferation and differentiation under acute conditions, they may also contribute to neurodegeneration when their activity becomes chronic. Neuronal differentiation can be promoted through the MAPK/CREB pathway through gp130-dependent signalling, which is activated by the IL-6/sIL-6R (Hyper-IL-6) complex. Neuronal differentiation in cortical neural progenitor cells is promoted through the non-canonical Wnt5 a/RhoA/ROCK/JNK pathway by IL-1 β. Despite this, persistent inflammation and elevated levels of IFN-*γ* and TNF could hinder the migration and differentiation timing of NSCs. Similarly, persistent activation leads to excessive production of IL-1β in NSCs which limits synaptic repair, while chronic exposure to IFN-*α* reduces the number of proliferating NSCs and neurons. TNF signalling is crucial in maintaining NSCs pool homeostasis and transitioning from activation to quiescence. Homeostasis, the processes of neuroprotection, neurogenesis and neural recovery can be supported by anti-inflammatory cytokines, which are highlighted in the section on the right. IL-4 is crucial for restoring the plasticity of NSCs and mediates the shift of macrophages and microglia toward an anti-inflammatory phenotype, suppressing the activation of the NF-κB/p65 pathway. IL-10 and TGF-β 1 transduction are involved in modulating neuroinflammation by up-regulating TREM-2, which aids in neuronal survival and axon-regeneration. The final fate of NSCs is determined by the interaction between pro-inflammatory and anti-inflammatory cytokines, which influences their ability to mediate neurological repair following traumatic or ischemic.

Collectively, these findings highlight the convergent role of immunomodulatory interventions in modulating neuroinflammation and supporting neurodegeneration across different models of neurodegenerative and neurotraumatic diseases (Figure 2).

### 3.3 Interplay between neuroinflammation and redox signalling in NSCs fate

Neuroinflammation is characterized by a redox imbalance that undermines protective inflammatory responses and drives chronic, homeostasis-disrupting processes (Adamu et al., 2024). Neuroinflammation and oxidative stress establish a self-reinforcing loop that intersects with key morphogenic pathways regulating NSCs fate, such as Notch, Wnt and Shh signaling. However, a direct causal impact of Notch under oxidative stress on NSCs fate decisions has yet to be conclusively demonstrated and, where reported, is largely limited to the cancer context (Chiappara et al., 2024). Wnt signaling has been shown to depend on ROS to activate neurogenic transcriptional programs. In NSCs, depletion of growth factors triggers cytoplasmic Ca^2+^ release followed by mitochondrial Ca^2+^ uptake, which enhances mitochondrial ROS production. The increase of ROS determines the oxidation of nucleoredoxin (NRX), a redox-sensitive inhibitor of the Wnt pathway. NRX oxidation releases Disheveled, a key activator of canonical Wnt signaling permitting β-catenin stabilization and the transcription of neurogenic genes that promote neuronal differentiation. However, when ROS levels exceed physiological thresholds, this redox-dependent activation of Wnt signaling becomes disrupted (Rharass et al., 2014). Excessive ROS destabilize essential Wnt components and impair β-catenin–mediated transcription, ultimately leading to defective neurogenesis and cognitive dysfunction. Together, these findings underscore a tightly interconnected regulatory axis in which ROS–Wnt crosstalk plays a critical role in guiding NSCs differentiation (Chatterjee and Sil, 2022). Moreover, under conditions of elevated ROS, Shh preserves neuronal viability and promotes neurite growth by stabilizing mitochondrial function, restoring membrane potential and ATP production, and enhancing endogenous antioxidant defenses (He et al., 2017). Although these effects were demonstrated in differentiated cortical neurons, they can be highly relevant to NSCs, which rely on Shh signaling supporting neurogenesis by maintaining redox balance, protecting mitochondrial integrity, and enabling the maturation and neurite extension of newly generated neurons (Favaro et al., 2009).

Thus, ROS–morphogen interactions within NSCs cannot be separated from the broader inflammatory environment, where microglia act as dominant ROS producers that directly modulate NSCs fate decisions. As the resident immune cells of the CNS and key guardians of brain homeostasis, microglia play a central role in neuroinflammation through their activation. Microglia, when activated by via damage-associated molecular patterns such as Aβ, lipopolysaccharide (LPS) or cytokines, rapidly activate enzymatic systems, especially NADPH oxidase (NOX), that generate ROS. NOX-derived ROS are the main reactive source during the inflammatory microglial response. After binding to pattern recognition receptor intracellular signals activate kinases, such as MAPK/p38/ERK that induce the translocation of NOX subunits to the membrane and the production of superoxide. This production is initially protective but in chronic conditions it becomes pathogenic, contributing to neuronal damage and the progression of neurodegeneration (Simpson and Oliver, 2020). The contribution of ROS to neuroinflammatory pathogenesis is well documented across major neurodegenerative diseases, where dysregulated inflammatory signaling and oxidative stress frequently coexist (Zhang et al., 2023). ROS production, especially from NOX4, contributes to the activation of the NLRP3 inflammasome, a central complex in neuroinflammation which is known to be implicated in tauopathy in Alzheimer’s disease (AD) (Ising et al., 2019). NSCs regulate NLRP3 activation and IL-1β secretion, which are critical in the initiation of the inflammatory responses, hence preventing the release of neurotoxic pro-inflammatory factors by microglia (Chan et al., 2019). In conditions of neuroinflammation and oxidative stress, microglial signalling profoundly influences NSCs differentiation and their recruitment to sites of damage, helping to shape a microenvironment that may support or hinder regeneration (Cavalcanti et al., 2025). Notably, a pro-inflammatory microglial phenotype neurogenesis and promotes astrogliosis, promoting the caspase-1 and the release of IL-1β and IL-18, amplifying and aggravating neuronal damage (Chen et al., 2020). Furthermore, like microglia, astrocytes also express NOX in response to LPS, INF-*γ* and Aβ contributing to neuronal damage (Vay et al., 2018). ROS can activate several intracellular pathways that promote astrogliosis such as NF-κB, p38/MAPK and JAK/STAT that are linked to NSCs fate. Indeed pharmacological inhibition of NF-κB abolishes proliferation of NSCs induced by TNF (Widera et al., 2006). The enhanced activity of the p38/MAPK in the *Atm*^−/−^ NSCs has been associated with intrinsic elevation of ROS limiting NSCs proliferation (Kim and Wong, 2009); cytokines activate JAK/STAT3 leading to proliferation and migration of NSCs to the spinal cord lesion, where they differentiate exclusively into astrocytes (Wang et al., 2015). Increased ROS production has been associated with the loss of dopaminergic neurons in Parkinson’s disease (PD) (Teleanu et al., 2022). The molecular mechanisms underlying this neuronal loss are linked to mitochondrial dysfunctions that increase ROS levels, which can interfere with the enzymatic processes of oxidative deamination of dopamine. This generates reactive intermediates that can increase oxidative stress including 6-hydroxydopamine and R-Salsolinol and participate in Fenton reaction to generate damaging hydroxyl radicals (Trist et al., 2019). The dopamine D1 receptor expressed in NSCs play a crucial role in maintaining redox and metabolic homeostasis. Indeed, it has been reported that hexokinase activity at the mitochondrial level can interfere with the modulation of ROS by dopamine stimulation, leading to a reduced mitochondrial calcium uptake in NSCs (Trist et al., 2019). These findings identify hexokinase as a dopamine-sensitive of mitochondrial ROS and Ca^2+^, suggesting relevance for PD and highlighting hexokinase and dopamine signalling as a potential vulnerability and therapeutic target, but direct evidence in PD models and human dopaminergic neurons are still needed. Oxidative stress represents one of the central factors that trigger and amplify the pathogenic mechanisms of Amyotrophic lateral sclerosis (ALS) (Cunha-Oliveira et al., 2020); as in PD, ALS mitochondrial and calcium regulation dysfunction can be an hallmark, which further amplifies the production of ROS (Zhou et al., 2019). In particular, at the presynaptic terminals of motor neurons, ROS can inhibit the release of acetylcholine, affecting neuromuscular transmission and altering the function of Soluble N-ethylmaleimide-sensitive factor attachment protein receptors (SNARE) protein (Pollari et al., 2014). Studies of PD-related genes in NSCs have suggested, have suggested that alteration of these genes are associated with the deregulation of proliferation, maintenance and differentiation (Le Grand et al., 2015). Many neurodegenerative disorders, including AD, PD, ALS, and traumatic injuries, feature synaptic and axonal transport dysregulation (Brunden et al., 2017). It can be appropriate to target the dysregulated signaling pathway to facilitate NSCs with neuronal maturation and axonal connectivity in response to injury. Proper axonal transport is critical to the normal functioning of neurons, and impairments in this process could contribute to microtubules stability. Neuronal integrity can be altered by ROS also at the level of specific microtubule components such as end-binding protein 1 (EB1), where a reduction in antioxidant systems such as superoxide dismutase and glutathione determines axonal swellings and microtubule disorganization in brains from aged Drosophila (Shields et al., 2024). Proper microtubule function is key to regulating essential processes for neurogenesis, such as cell division, migration, polarization, and neurite outgrowth. Their dynamics have been found to be finely modulated by post-translational modifications of tubulin by the acetyltransferase GCN5/KAT2A, maintaining axonal growth and proper differentiation during NSCs neurogenesis (Lin et al., 2022). ROS-mediated oxidative stress is regarded as a key factor in AD, given its strong link to Aβ accumulation and deposition. Recent evidence reveals that mitochondria in neurogenic niches do not only respond to but also propagate neuroinflammatory factors. In neurodegenerative diseases, mitochondrial fusion/fission imbalance, elevated ROS and disturbed Ca^2+^ handling are intimately linked with microglial activation and cytokine release (de Oliveira et al., 2021; Qin et al., 2024). Mitochondrial deregulations in AD determine an increase in ROS with consequences associated with energy loss, cell death and hyperphosphorylation of tau for the accumulation of neurofibrillary tangles. In AD, the alteration of calcium homeostasis at mitochondrial level can increase the production of ROS including also the dysregulation of mitochondrial fission and fusion dynamics (Misrani et al., 2021). In this regard, NSCs are not only influenced by mitochondrial dynamics, but actively shape them, using mitochondrial remodeling. According to the studies discussed in the review by Laaper et al., the balance between fusion and fission constitutes a primary regulator of NSCs fate decisions (Laaper and Jahani-Asl, 2018). Indeed, blocking the fusion proteins Mfn1/2 in NSCs leads to learning and memory deficits, demonstrating that mitochondrial dynamics in NSCs function not only as intrinsic cellular regulators but also influence cognitive processes across the brain. These findings highlight how dysregulation in NSCs mitochondrial homeostasis can generate pathophysiological consequences that may significantly contribute to disorders characterized by impaired memory circuits, such as AD (Khacho et al., 2016). Overall, the interplay between inflammatory mediators, ROS accumulation, and mitochondrial imbalance underscores the potential effects on NSCs within a chronically inflamed niche.

## 4 Oxidative stress responses and NSCs fate crosstalk

Redox regulation represents a fundamental cellular process in which non-toxic levels of ROS, generated through redox reactions, can act as second messengers, promoting physiological cellular processes (Le Belle et al., 2011). Several lines of evidence indicate that ROS balance maintains NSCs homeostasis for stemness, proliferation and neurogenesis through various redox sensing proteins. High or prolonged levels of ROS induce oxidative stress, apoptosis, or premature differentiation, reducing stem cell reserve (Hwang et al., 2021). Neuroinflammatory conditions trigger stress-related cellular changes that lead to increased ROS levels, which are toxic to cell survival. The proper balance of ROS and the resulting signalling has also been linked to cellular developmental processes (Bigarella et al., 2014; Reczek and Chandel, 2015). Collectively, several evidence indicate that ROS regulate NSCs fate in a hormetic, dose-dependent manner: low levels stimulate proliferation and differentiation, whereas high concentrations trigger senescence, exhaustion, and cell death (Abdal Dayem et al., 2022; Chaudhari et al., 2014). However, the dose-dependent concept is extremely simplified in the biological complexity that must be considered in NSCs under neuroinflammatory conditions. Physiological levels of ROS contribute to the proper spatiotemporal organization of multicellular systems. For example, studies on *Caenorhabditis elegans* have shown a temporal production of ROS during the larval stage, followed by a decreased level in adults (Miranda-Vizuete and Veal, 2017). Other studies on animal models have reported spatio-temporal correlations between ROS and tissue regeneration with the activation of redox signalling system (Jones and Sies, 2015). For this reason, it is important to understand the shift of ROS in neuroinflammatory conditions and their impact on NSCs homeostasis.

### 4.1 Key molecular pathways ROS-induced in NSCs

#### 4.1.1 NRF2 signalling

Nuclear factor erythroid 2-related factor 2 (NRF2) has emerged as it represents the master regulator of the cellular defense against oxidative stress. In this context, NRF2 dissociates from its binding to the negative regulator Kelch-like ECH-associated protein 1 (KEAP1), activating the transcription of several genes through the interaction with their antioxidant response elements (AREs) (Sparaneo et al., 2025). ROS-NRF2 levels must be carefully balanced during cell development, coordinating phases of NSCs quiescence, in which cells rely on glycolytic metabolism, with phases of NSCs activity and differentiation, where the switch to oxidative phosphorylation (OXPHOS) requires tighter control of ROS and consequent activation of NRF2 (Dodson et al., 2021). Specific transcription factors downstream of NRF2 that regulate NSCs are still to be elucidated, although its key role in NRF2 has been demonstrated in adult *Nrf2^−/−^* mice. In this model, an impaired long-term potentiation, a lower proliferative and clonogenic capacity of neutrosphere have been associated with reduction of doublecortin positive cell as compared to *Nrf2*^+/+^ mice (Robledinos-Anton et al., 2017). Studies exploring molecular mechanisms remain relatively few and it is still not clear how Keap1-NRF2 dissociation is deregulated in NSCs from physiological to pathological conditions such as neuroinflammation. However, it has been reported that NSCs derived from Huntington’s disease patients do not respond to MIND4-17, a compound that activates the NRF2 pathway by modifying the C151 cysteine residue on Keap1. This lack of responsiveness suggests that Keap1 modifications induced under chronic stress and neuroinflammatory conditions may alter NRF2 pathway activation in these cells (Quinti et al., 2017). A second work showed that the activation of NSCs derived from spinal cord models was suppressed after pro-inflammatory signal of complement C3a, inducing Nrf2 ubiquitination and degradation. This was associated to NF-κB p65 pathway through the deubiquitinating enzyme ubiquitin carboxy-terminal hydrolase L1 (UCHL1)-proteasome system. These effects resulted in increased NSC deficiency and therefore a reduced repair response (Ding et al., 2025).

Correlations between ROS-NRF2 axis and stemness are not limited to NSCs but also to other cell types that may share stemness transcriptional programs with NSCs. This knowledge could have a significant impact on those tissues or organs that are exposed to transplants for neurogenerative purposes, such as in the case of hepatic progenitor cells, which thrive in microenvironments where acute and chronic inflammation leads to ROS exposure (di Bello et al., 2018). Indeed, the potential key role of NRF2 in the modulation of metabolism and redox systems has been reported in several stem cell lines (Dai et al., 2020). NRF2-dependent control of NSCs regeneration influences functional neurogenesis-related behaviors, in tasks like the Morris water maze and pattern separation tests, identifying a critical time in middle-aged rats (13–15 months), when NRF2 expression is essential for NPCs regeneration and function (Limke and Rao, 2003). NRF2 also influences inflammatory pathways, such as the NF-κB pathway, which can be relevant in the context of neurodegenerative diseases, ensuring NSCs to maintain their stem-like properties and ability to produce new cells (Kahroba et al., 2021). Moreover, increased mitochondrial ROS, together with calcium influx in NSCs, can activate the Wnt/β-catenin signalling pathway that cooperate with NRF2 to regulate neuronal differentiation (Rharass et al., 2014; Marchetti, 2020); although detailed molecular evidence is lacking, it is also shown that NRF2 acts upstream to preserve the molecular and metabolic conditions required for canonical Wnt signaling to function in NSCs that can be altered during neuroinflammation (L'Episcopo et al., 2013); a reduction in NRF2-induced AREs such as heme oxygenase 1, triggers inflammatory factors promoting NF-κB. It activates inflammatory mediators from microglia that converge to dysregulate the Wnt/Fzd/β-catenin pathway. Mechanistically, this deregulation is linked to impaired PI3K/Akt activity and aberrant GSK-3β regulation. Because PI3K/Akt normally inhibits GSK-3β, NRF2 reduction leads to loss of PI3K/Akt activation, permitting GSK-3β to accelerate β-catenin degradation and attenuates canonical Wnt signaling (L'Episcopo et al., 2013). In NSCs, this may result in diminished proliferative capacity and impaired neurogenic process, clarifying how NRF2 dysfunction compromises Wnt-dependent stem cell fate.

NRF2 downregulation has been identified as an important contributor for NSCs survival and regeneration decline in rats around 13–15 months. NRF2 reduction and NSCs deterioration has been associated with the loss of ability to counter oxidative and metabolic stress, leading to functional exhaustion of the NSCs. Therefore, NRF2 could have the potential to maintain balanced ROS levels or to support metabolic reprogramming for antioxidant defense, but whether NRF2 interacts with transcriptional regulation of specific neural stemness and differentiation markers has not been yet elucidated (Corenblum et al., 2016). Similarly, aging has been proposed to evaluate low-grade chronic inflammatory and oxidative environment affecting NSCs (Dodson et al., 2021; Signer and Morrison, 2013). *In vitro* knockdown NRF2 in dentate gyrus-derived NSCs have been shown to decrease their proliferation and survival, whereas *NRF2^−/−^* mice showed reduced minichromosomal maintenance complex component 2 (MCM2)-positive proliferative cells and fewer Sox2- and doublecortin (Dcx)-positive NSCs (Ray et al., 2018). NRF2 has been revealed as a central redox-sensitive transcription factor that regulates the balance between symmetric and asymmetric divisions in hippocampal SGZ. Under a balanced antioxidative state, NRF2 supports controlled NSCs-to-glial differentiation in wild-type mice, whereas in an oxidized redox environment it leads to premature depletion of the stem cell pool, as observed in *NRF2^−/−^* mice (Robledinos-Anton et al., 2017). This suggest that NRF2 operates in a stage-dependent manner, with NSCs requiring basal NRF2 activity to maintain quiescence and long-term self-renewal, while transient increases in NRF2 are necessary during activation and differentiation to sustain the metabolic energy and oxidative state that is required for lineage progression (Stoll et al., 2015). Other evidence for hippocampal neurogenesis sustained the role of NRF2 in NSCs recovery of cognitive impairment synergistically with mitochondrial complex I inhibition by metformin (Fan et al., 2023). Although direct NRF2 activation by metformin has not been demonstrated, previous studies have been reported that an increased AMP/ATP ratio activates AMPK. AMPK, in turn, promotes NRF2 activation through inhibition of GSK3β inhibition, facilitating its nuclear translocation and the transcription of antioxidant genes such as HO-1, NQO1, and G6PD, thereby restoring redox balance (Joo et al., 2016). NRF2 can interact with several signalling pathways that influence neurogenesis and cell survival. NRF2 crosstalk with mTOR involves p21 as a key mediator through inhibition of Keap1, which stabilized NRF2 (Zoungrana et al., 2022). The observation that mTOR can enhance NRF2 stability via p21-mediated Keap1 inhibition implies that NSCs use this axis to couple growth factors to redox protection and survival (Urban et al., 2019). Moreover, the presence of ARE within the mTOR promoter establishes a direct transcriptional link between NRF2 and mTOR (Gureev et al., 2020). This means that NRF2 is not only a downstream factor of mTOR (via p21-mediated Keap1 inhibition) but also an upstream transcriptional regulator of mTOR expression. Fluctuations in NRF2 levels, whether driven by redox status, inflammation, or aging, can directly modulate mTOR expression. In NSCs, where mTOR governs quiescence, activation, proliferation, and differentiation, the link with NRF2 signaling may create a mechanism through which redox conditions feed into growth-control circuitry (Paliouras et al., 2012). NRF2 also protects NSCs from Aβ toxicity, and its activity is linked to the PI3K/Akt and MEK/ERK1/2 pathways in controlling neurogenic process, although its precise role during specific stages of differentiation under stress condition remains unclear (Karkkainen et al., 2014; Boorman et al., 2022).

#### 4.1.2 NRF2/notch axis

The NRF2–Notch signalling interplay forms a reciprocal regulatory loop that integrates redox homeostasis with cell fate control, providing a molecular framework for maintaining stemness under stress conditions. Experimental evidence indicates that NRF2 directly activates Notch1 transcription through AREs located within the Notch1 promoter, while the Notch intracellular domain (NICD) binds to conserved Rbpjκ sites in the NRF2 promoter, driving its transcription (Kahroba et al., 2021). NRF2-mediated Notch signalling improves hematopoietic stem cell function after myelosuppression induced by ionizing radiation (Kim et al., 2014). The study by Wakabayashi et al. reveals a direct molecular connection between Notch and NRF2 signalling, forming a bidirectional regulatory circuit that could play a crucial role in sustaining stem cell identity under oxidative and metabolic stress. Constitutive NICD expression leads to upregulation of NRF2 and its cytoprotective target genes, enhancing resistance to oxidative and xenobiotic stress (Wakabayashi et al., 2014). *In vivo*, NRF2 deficiency delayed liver regeneration after partial hepatectomy, a defect fully rescued by reintroducing the active domain NICD in hepatocytes (Wakabayashi et al., 2010). KEAP1 silencing also upregulated Notch1 and its target HES1, modulating DLL3, a known neuroendocrine differentiation marker mirroring mechanisms active in stem cells (Fabrizio et al., 2024; Sparaneo et al., 2025). This establishes a bidirectional regulatory circuit in which each pathway amplifies the other’s activity. Such mutual reinforcement ensures synchronized regulation of stress responses and cell renewal, especially in contexts requiring regeneration or repair. In NSCs, NRF2 activity sustains a redox environment conducive to self-renewal, protecting against deregulated differentiation or apoptosis ROS-induced and representing a druggable target for neuroinflammatory conditions (Kahroba et al., 2021). Loss of key mitochondrial fusion proteins increased ROS in NSCs, leading to NRF2 activation that induced the upregulation Botch, a known inhibitor of Notch signaling, favoring the exit from the active quiescence state and promoting the initiation of the neurogenic program. The genetic deletion of NRF2, abolished the induction of Botch and restored self-renewal capacity in mitofusin-deficient NSCs (Khacho et al., 2016). These processes were supported by further RNA-seq analyses showed that NRF2 controls a coordinated transcriptional network including both Botch, which attenuates Notch-dependent self-renewal and activated pro-neuronal genes (Khacho et al., 2016). These evidence support the stage-dependent function of NRF2, whereby basal NRF2 activity supports NSCs survival and self-renewal, while transient NRF2 activation promotes differentiation through Botch-mediated attenuation of Notch signaling. However, several lines of evidence support a functional role for NRF2 in NSCs neurogenesis, although detailed mechanistic studies on morphogenic pathways such as Notch are still limited (Dodson et al., 2021; Boorman et al., 2022). NRF2 can directly regulate the Notch1 promoter and Notch target genes (Hes1, Hey1) in non-neural tissues that can plausibly extend to NSCs (Wakabayashi et al., 2015). Considering the central role of Notch in controlling the fate of NSCs, further investigations for NRF2–Notch axis can reveal homeostatic regulatory processes under neuroinflammatory and redox signals.

#### 4.1.3 PI3K/Akt and FOXO signalling

Redox status influences NSC biology through a tightly interconnected network of PI3K/AKT, MAPK, and NOX-dependent signaling mechanisms. It has been reported that PI3K/AKT/mTOR is a major pathway that is regulated by redox status through the oxidative inactivation of PTEN, leading to signalling and proliferation of adult hippocampal stem/progenitor cells (Dickinson et al., 2011). Indeed, ROS can inhibit PTEN, increasing the activation of the PI3K/Akt pathway, which is essential for NSCs function (Hwang et al., 2021). However, ROS-dependent modulation of these pathways can have divergent outcomes depending on cellular context. For example, elevated ROS in mice lacking Atm, activate the p38 MAPK pathway, leading to impaired self-renewal, whereas low ROS in Akt1/2 double-knockout mice compromise the differentiation capacity of hematopoietic stem cells, demonstrating that both insufficient and excessive oxidative signaling can disrupt stem cell function (Ito et al., 2006; Juntilla et al., 2010). Moreover, the self-renewal and neurogenic potential of NSCs are stimulated by NADPH oxidase (NOX)–derived ROS in undifferentiated cells, but not during differentiation (Le Belle et al., 2011). Similarly, 2-acetylacteoside enhanced the expression of phosphorylated Akt in cultured NSCs after oxygen–glucose deprivation/reoxygenation, promoting neurogenesis (Wang et al., 2024). The proliferation of NSCs in the SVZ via the NOX2-ROS-PI3K/Akt pathway has been induced by chemokine CXCL1, increasing ROS production (Shang et al., 2019). ROS signaling can also bias NSCs toward differentiation rather than proliferation. Indeed, an increase in ROS mediated by PI3K-AKT signalling was associated with neuronal differentiation of NSCs after formyl peptide receptors agonist (Zhang et al., 2017). PI3K/Akt and MAPK signalling activation enhanced hippocampal neurogenesis increasing ROS in Ferroportin 1 conditional knockout mice (Ding et al., 2025). Additional involvement of MAPK pathways has been described in D-gal-induced senescence of NSCs derived from human embryonic stem cells, underscoring how redox-sensitive MAPK signaling intersects with aging-associated stress responses (Pan et al., 2025). Collectively, these findings indicate that ROS do not exert a unidirectional effect on NSCs which can perform their function as modulators driving proliferation, differentiation, or promote a decline depending on the balance between PI3K/AKT, MAPK, and NOX-derived signaling. Understanding how these pathways integrate redox cues is essential for clarifying NSCs fate under neuroinflammatory and oxidative stress conditions.

Forkhead box O (FOXO) transcription factors integrate oxidative signals with the transcriptional control of stem cell maintenance through redox-sensitive networks that stabilize metabolism and preserve stemness. Comparable to NRF2, increased oxidative stress can promote differentiation, while FOXO activation adjusts stemness-related genes expression and antioxidant defenses in a stem cell type–specific manner (Soh et al., 2021). FOXO1 and FOXO3 expression is high in quiescent Sox2^+^ NSCs and is decreased upon activation and differentiation. Quiescent NSCs reside in a mildly hypoxic niche and are primarily glycolytic, which supports self-renewal by limiting mitochondrial activity and ROS production. FOXO factors maintain this state by regulating cell-cycle genes and controlling glucose and glutamine metabolism, thereby preventing premature proliferation and exhaustion of the NSCs pool. When FOXO activity decreases in activated NSCs, metabolism shift toward lipogenesis and oxidative phosphorylation (OXPHOS), leading to ROS signalling that promotes neuronal differentiation (Ludikhuize and Rodriguez Colman, 2021).

Interconnected relationship between FOXO signalling and molecular pathway under redox stress was also found for Hippo protein kinase; the coordination between the Hippo pathway and redox signalling ensures proper regulation of biological processes involved in organ development (Zheng et al., 2020). Under oxidative conditions, this coupling can shift Hippo output toward pro-apoptotic programs via MST/YAP/FOXO signaling, highlighting how redox stress redirects pathways normally involved in tissue homeostasis and organ development (Zeybek et al., 2021). Within the NSCs niche, this redox–Hippo axis intersects with Wnt signaling through the downstream transcriptional cofactor TAZ, which is highly expressed in Nestin^+^ cells in both SGZ and SVZ and naturally declines with age disappearing in DCX^+^ immature neurons. Age-related reductions in NRF2 activity further diminish TAZ levels, contributing to decreased NSC self-renewal and altered transcriptional dynamics. Indeed, as NRF2 activity declines with age, TAZ levels decrease and NSCs reduce self-renewal, inducing TAZ to form a functional complex with TEAD1–4. Bioinformatics and ChIP-qPCR have identified TAZ/TEAD binding sites in the promoters of Sox2, Ascl1, Neurog2, and NeuroD1, indicating that the redox-dependent modulation of the Hippo–Wnt–FOXO axis has direct consequences for the maintenance and neurogenic potential of NSCs (Robledinos-Anton et al., 2020).

#### 4.1.4 Metabolic reprogramming and mitochondrial dynamics alteration

Molecular pathways associated with oxidative stress can have a significant impact on the NSCs metabolic configuration. The metabolic adaptations of NSCs to oxidative stress, which involve metabolic reprogramming, may be accompanied by changes that influence NSCs fate.

Within the framework of redox regulation, mitochondria represent central hubs of cellular energy metabolism and redox signalling, acting as both the main producers of ATP and a key source of ROS (Khacho and Slack, 2018). Accumulating evidence highlights a tight coupling between cellular metabolism and redox homeostasis, which acts as a key determinant of NSCs functions.

As previously mentioned for NRF2 regulation, a predominant reliance on glycolysis over mitochondrial OXPHOS for energy demands supports NSCs stemness by relatively lower mitochondrial ATP production, reduced ROS production, and enhanced antioxidant capacity, which are critical for protecting stem cell genomic integrity and preventing premature differentiation (Zoccarato et al., 2022). As NSCs commit to neuronal or glial lineages, they undergo metabolic reprogramming, switching from glycolysis to OXPHOS with ROS production acting as signalling molecules that promote differentiation-related transcription programs. Recent evidence has uncovered link between cytoskeletal integrity and mitochondrial metabolism in NSCs (Garone et al., 2024). Indeed NESTIN–cyclin-dependent kinase 5–DRP1 regulatory axis has been identified as a key determinant of mitochondrial morphology and energy metabolism in NPCs, acting as a negative regulator of OXPHOS (Wang et al., 2018). Indeed, under control of the cytosolic cyclin-dependent kinase 5, DRP1 is phosphorylated leading to mitochondrial fission. However, DRP1 exhibited a low activation following *NESTIN* knockdown linked to Cdk5 depletion (Wang et al., 2018). Mitochondrial structural proteins have been studied in AD patients at different stage reporting that Aβ interacts with DRP1 altering mitochondrial dynamics and increasing during AD progression (Manczak et al., 2011).

Mitochondrial fragmentation, ROS accumulation, and impaired energy metabolism resulted in oxidative stress with elevated lipid peroxidation and decreased activity of antioxidant enzymes, driving NSCs apoptosis and biases differentiation toward astroglial lineages (Ribeiro et al., 2019). Mitochondrial dynamics are closely linked to the functional state of NSCs, adapting from quiescence to activation through the coordinated action of specific regulators. ATP-dependent mitochondrial protease YME1L activity is dynamically regulated during transitions between quiescent and active NSCs states, thereby enabling remodeling of mitochondrial proteome to meet the metabolic demands of each phase. The absence of YME1L activity leads to defective mitochondrial quality control, metabolic remodeling failure, and altered mitochondrial morphology. As a consequence, NSCs exhibit premature differentiation and progressive stem cell pool depletion (Wani et al., 2022). Beyond metabolic and structural regulators, stress-responsive kinases, such as JNK, play crucial roles in preserving mitochondrial function in NSCs. Indeed, JNK inhibition leads to elevated mitochondrial ROS production, depolarization of the mitochondrial membrane, and reduced NSC survival, linking kinase signalling to the maintenance of redox homeostasis and mitochondrial dynamics (Sharma et al., 2018).

The interplay between redox signalling and mitochondrial function of NSCs has been investigated using various antioxidant agents. For instance, inhibition of Wnt/β-catenin signalling has been associated with increased oxidative stress and apoptosis in NSCs (Zhao et al., 2018). Moreover, pre-treatment of NSCs with N-acetylcysteine markedly attenuates paraquat-induced toxicity, preventing mitochondrial fragmentation, reducing autophagic activity, and enhancing the expression of anti-apoptotic proteins (Xiong et al., 2019). This interplay between antioxidant defense and metabolic regulation reflects a broader adaptive mechanism also observed with natural compounds such as *Quadrella incana*, whose extract enhances redox homeostasis by activating FoxO1, a transcription factor pivotal for redox regulation that induces expression of its antioxidant targets, such as PRDX3 and SOD2, which are key mitochondrial and cytosolic ROS scavengers, respectively. In addition, it has been reported an upregulation of glycolytic genes including *GLUT1, GLUT3,* and *LDHa* sustaining glycolytic metabolism for preserving NSCs undifferentiated state (Kang et al., 2020).

**Figure 3:**
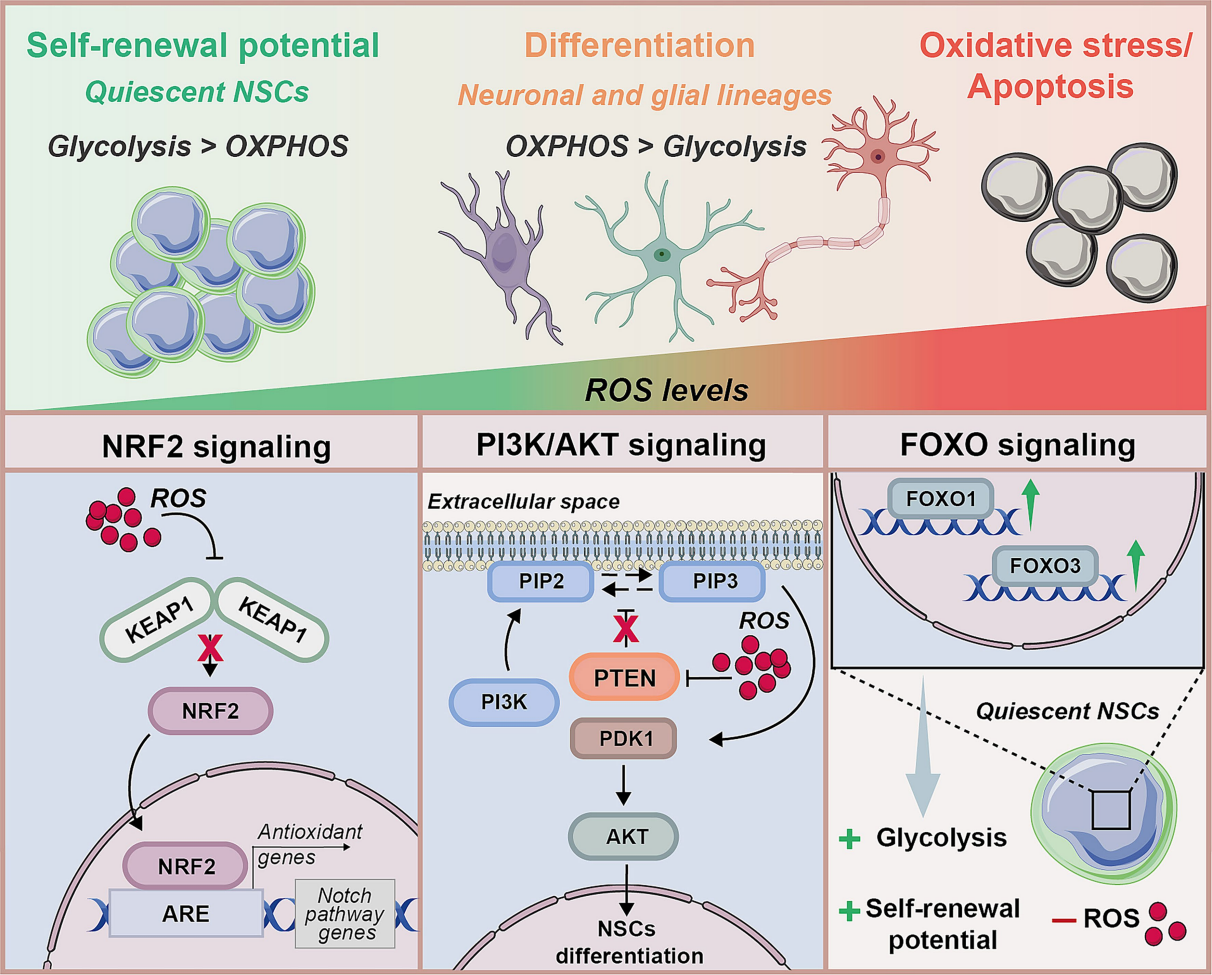
Reactive oxygen species (ROS) act as key players of NSCs behavior by influencing the balance between self-renewal, differentiation, and oxidative stress-induced apoptosis. Low ROS levels support the maintenance of quiescent NSCs with high self-renewal potential and a glycolysis-dominant metabolic profile, whereas moderate ROS levels promote differentiation toward neuronal and glial lineages, with a metabolic shift to oxidative phosphorylation (OXPHOS). High ROS levels lead to oxidative stress and cell death. Three major redox-sensitive pathways coordinate these processes: (i) NRF2 signalling, in which ROS inhibit KEAP1, blocking NRF2 nuclear translocation and preventing the activation of antioxidant response elements (ARE). It depicts signaling pathways: NRF2-Notch axis forms a reciprocal regulatory loop: NICD binds the NFE2L2 promoter to enhance NRF2 transcription, while NRF2 upregulates Notch and related components by binding AREs within their promoters (ii) PI3K/AKT signalling regulates NSCs differentiation through PI3K-mediated AKT activation; ROS negatively modulate the phosphatase PTEN, blocking its mechanism and activating AKT, that leads to an increase in NSCs differentiation; (iii) FOXO signalling, with FOXO1 and FOXO3 expression higher in quiescent NSCs, promotes glycolytic metabolism and NSCs self-renewal potential, limiting ROS production.

Together, these findings illustrate that NSCs fate is critically governed by the interplay between mitochondrial dynamics, metabolic reprogramming, and redox regulation. Mitochondria not only supply energy but also function as redox signalling hubs, translating metabolic status into fate-determining signals (Figure 3). The transition from glycolysis to OXPHOS during differentiation elevates ROS production, which, when properly buffered, acts as a physiological induction for lineage specification. Accumulated evidence underscore that redox control is not merely protective but instructive, guiding NSCs transitions between quiescence, activation, and differentiation through tightly coupled metabolic and mitochondrial mechanisms. Disruption of this equilibrium, by genetic, metabolic, or inflammatory stressors, compromises neurogenesis and stem cell maintenance, highlighting mitochondrial–redox crosstalk as a fundamental determinant of NSCs fate and regenerative potential.

## 5 Conclusion and perspectives

NSCs can be embraced in a dynamic interface between neurogenesis, tissue repair, and the pathophysiological challenges imposed by inflammation and oxidative stress. Across developmental and adult stages, their ability to self-renew, differentiation, and modulation of the surrounding microenvironment depends on a delicate equilibrium between redox homeostasis, inflammatory signalling, and metabolic adaptation. Inflammation and oxidative stress play key roles in shaping NSCs fate, which are not exclusively detrimental for their cellular functions. NSCs represent both sensors and modulators of the neuroinflammatory environment, responding to immunological factors through pathways that can either promote repair or exacerbate degeneration, depending on the timing and intensity, which should be further investigated. Their secretory and immunomodulatory properties highlight their potential as therapeutic agents to counteract neuroinflammation and to promote regeneration.

At the molecular level, the transcription factor NRF2 is central to this regulation, acting as the master coordinator of antioxidant responses. Through its KEAP1–ARE regulatory axis, NRF2 preserves NSCs viability and redox stability, supports metabolic plasticity and regenerative functions. Furthermore, its reciprocal interaction with Notch signalling establishes an NRF2–Notch axis that links redox balance to NSCs homeostasis. This bidirectional regulatory loop may act as a hot spot ensuring proper transitions of NSCs between quiescence, proliferation, and differentiation under oxidative or inflammatory stress. NSCs fate comprise several redox-sensitive signalling cascades, including PI3K/Akt, MAPK, and Wnt/β-catenin, which collectively regulate stemness, proliferation, and lineage specification. The balance between reactive ROS production and antioxidant defense defines whether these pathways promote neurogenesis or trigger senescence and apoptosis.

This review also highlights the crucial contribution of molecular pathways integrating redox and metabolic signals that can be fostered in inflammatory conditions to sustain quiescence, prevent excessive ROS accumulation, and maintain NSCs integrity. These transcriptional networks are tightly coupled with mitochondrial dynamics and metabolic reprogramming: glycolytic metabolism supports the quiescent, stem-like state, whereas the shift toward oxidative phosphorylation promotes differentiation through controlled ROS signalling. Disruption of this redox and metabolic coupling by chronic inflammation, aging, or mitochondrial dysfunction, drives premature differentiation and depletion of NSCs reservoir.

Taken together, current evidence defines oxidative stress and inflammatory signalling as dual regulators of NSCs fate, capable of orchestrating repair when finely regulated, or destructive when uncontrolled. Future therapeutic strategies should exploit this balance, enhancing stress response pathway and metabolic adaptive strategies to understand NSCs lineage and function during oxidative stress and neuroinflammation. Such approaches could open new avenues for treating neurodegenerative diseases, brain injury, and other CNS disorders by reinforcing the intrinsic regenerative capacity of NSCs.

### 5.1 Translational relevance of NSC-based therapy

The regenerative potential of NSCs has attracted considerable attention as a therapeutic strategy for neurodegenerative diseases and pathologies of CNS, also including trauma and ischemic injury (Zhou et al., 2019). Recent reviews have examined the therapeutic relevance of NSCs in neurodegeneration, highlighting both preclinical advances and clinical trials (Lutfi Ismaeel et al., 2023). Notably, NSC-based interventions have also been investigated in the context of tumors arising from dysregulated NSCs programs, such as gliomas, primarily as vehicles for targeted drug delivery (Fawzy et al., 2025). Among the proposed strategies, direct NSCs transplantation remains the most extensively pursued approach; however, despite notable progress, it still faces substantial limitations in reliability, reproducibility, and long-term functional integration (Kaneko et al., 2011). Key studies discussed in the review, that examine how neuroinflammation and oxidative stress can be linked across major neurodegenerative diseases, with particular attention to the potential involvement of NSCs, are summarized in Table 1. A major critical issue for clinical translation lies in the incomplete understanding of NSCs differentiation, migration, and maturation under both physio-pathological conditions. In particular, the molecular mechanisms by which NSCs respond to neuroinflammatory factors and oxidative stress remain insufficiently defined. This gap is critical, as neuroinflammation induces profound homeostatic disruptions that influence NSCs survival, lineage decisions, and reparative capacity. Beyond the technical challenges associated with NSCs isolation, expansion, and transplantation, the lack of mechanistic insight into how NSCs sense and adapt to inflammatory and redox stress represents a key barrier to therapeutic optimization. This knowledge gap also opens opportunities: elucidating redox-regulated pathways, endogenous antioxidant systems, and growth-factor-dependent protective mechanisms may enable the identification of therapeutic targets and NSC-specific biomarkers, particularly those reflecting mitochondrial dysfunction. Indeed, given that neuroinflammation and oxidative stress interfere with mitochondrial dynamics, a central determinant of NSCs metabolic state and fate, it may be advantageous to identify NSCs redox signatures associated to mitochondrial dysfunction aimed at guiding targeted interventions. Moreover, acting on NRF2 and other linked transcriptional program and signaling pathways, can prevent premature differentiation that reduces the regenerative pool enhance neurogenesis in chronic inflammatory diseases. In parallel, growing interest has been focused on the NSCs secretome, which includes neurotrophic and antioxidant factors with promising therapeutic potential (Yang et al., 2024). Moreover, the antioxidant capacity of NSC-derived extracellular vesicles, enriched in regulatory proteins, miRNAs, and potentially mitochondria, suggests an additional layer of metabolic and redox modulation that could be harnessed for therapy (Upadhya et al., 2020; Boukelmoune et al., 2018). In this regard, mitochondrial dysfunction and synaptic decline, driven by oxidative stress, that is commonly reported in neurodegenerative diseases, can be restored by acting on the trophic factors that are delivered by NSCs or by identifying the molecular pathways that lead to the efficiency of NSCs in their neurotrophic action. Identifying pathways that respond to oxidative stress in NSCs under these conditions may be useful for developing drug therapies that restore neurogenic function (Nath et al., 2024). NSCs are not only carriers of extracellular vesicles but can also be recipients of vesicle-derived material such as extracellular vesicles from microglia. In ischemia it has been observed that active microglia are able to produce vesicles containing miRNA-124 which determines the activation of Notch on NSCs mediated by adaptor-associated protein kinase 1 enhancing NSC proliferation and differentiation (Song et al., 2023). Further studies may reveal the role of the secretome in exchange between NSCs and microglia, proposing appropriate pharmacological interventions.

**Table 1:** Neurodegenerative diseases feature imbalanced pro- and anti-inflammatory signaling that affects NSCs fate by altering ROS homeostasis.

Disease	Biomarkers	NSCs involvement	Effects	References
AD	Pro-anti-inflammatory cytokines imbalance ↑ ROS	↑ Mitochondrial function and kinase signalling INF-γ induced	↑ Neurogenesis	Mastrangelo et al. (2009) and Baumann et al. (2020)
AD	↑ NLRP3/ROS	↑ Protective effects to reduce inflammatory response	↑ Tau hyperphosphorylation	Ising et al. (2019) and Chan et al. (2019)
AD	Pro-anti-inflammatory cytokines imbalance ↑ ROS	↓ Mitochondrial dynamics	↓ Mitochondrial Ca^2+^ homeostasis	de Oliveira et al. (2021)and Qin et al. (2024)
AD	↑ Kynurenic acid	↓ Neurogenic ability	↓ Regenerative capacity	Zhang et al. (2021)
PD	High hydroxyl radicals	↓ Mitochondrial Ca^2+^ uptake	↑ Neurotransmitters alteration	Assis-de-Lemos et al. (2021)
ALS	Pro-anti-inflammatory cytokines imbalance ↑ ROS	↑ Deregulation of proliferation, differentiation and maturation	↓ Neurotransmitters release	Pollari et al. (2014) and Le Grand et al. (2015)
ALS PD AD	Pro-anti-inflammatory cytokines imbalance ↑ ROS	↑ Altered microtubule dynamics	↑Alteration on axonal growth	Brunden et al. (2017) and Shields et al. (2024)
SCI	↓ SAM and SH3 domain-containing protein 1 (SASH1)	↑ Support BDNF release	↑ Recovery	Liu et al. (2023)
SCI	↑ TNF/Smac-mimetic/Z-VAD-FMK	↑ Necroptosis	↓ Recovery	Tong et al. (2023)
SNI	Pro-anti-inflammatory cytokines imbalance ↑ ROS	↓ P2 × 4R IL-1β and TNF ↓NF-KB signalling	↓ Recovery	Zhang et al. (2021)
SNI	Pro-anti-inflammatory cytokines imbalance ↑ ROS	↑ Protective effects to reduce inflammatory response	↑ Recovery	Chen et al. (2020)
Ischemia	Pro-anti-inflammatory cytokines imbalance ↑ ROS	↑ Neuronal differentiation INF-γ induced	↑ Neuronal repair	Zhang et al. (2018)
Ischemia	Increase TNF, IL-6/8, SOD, malondialdehyde	↑ GDNF/PI3K/AKT	↑ Recovery	Xu et al. (2023)
EAE	Low IL-10 / High IFN-γ and IL-17	↑ Protective effects to reduce inflammatory response TGFβ-induced	↑ Immunomodulatory capacity	Xie et al. (2018)

NSCs are vulnerable to the cytotoxic effects of chronic inflammation, which can compromise their capacity for homeostatic repair. Accordingly, many studies have focused on NSC transplantation to harness their immunomodulatory and regenerative properties. NSCs can enhance neurogenesis and tissue recovery through several mechanisms, including the protective actions of immunomodulatory cytokines and through the activation of pathways that stimulate the release of trophic factors such. However, under conditions of sustained neuroinflammation and oxidative stress, NSCs may instead exhibit disrupted mitochondrial dynamics, leading to altered calcium homeostasis, energy deficits, and impaired neurotransmitter synthesis and communication.

### 5.2 Conflicting evidence and knowledge gaps

Despite significant advances in understanding how neuroinflammation and oxidative stress influence NSCs fate, multiple areas remain marked by insufficient mechanistic resolution. NSCs themselves exhibit considerable heterogeneity, adopting distinct functional states or subtypes in response to inflammatory factors and activation of pathways (Murtaj et al., 2023). How the maturation or the stemness and singling pathways cooperate in neuroinflammatory conditions are incompletely defined, particularly given the extensive cross-talk among lineage-specific transcription factors and signaling cascades (Zhu et al., 2021). This interconnected regulatory network is not experimentally mapped, leading to divergent interpretations across studies. Likewise, the NRF2 pathway has been implicated in NSCs fate, but how Keap1-NRF2 dynamics are deregulated during neuroinflammation is not well understood. The broader relationship between NRF2, stemness programs, and other progenitor cell types adds additional complexity. A further area of debate concerns the dose-dependent effects of ROS. While a hormetic model, low ROS promoting proliferation and high ROS inducing senescence or death, is widely reported, this framework does not consider the highly context-dependent redox fluctuations occurring under neuroinflammatory conditions (Jomova et al., 2023). Interactions with cytokine signaling, mitochondrial dysfunction, and metabolic rewiring introduce variables that are rarely assessed simultaneously, contributing to inconsistent results across studies. Addressing these gaps, particularly those involving redox-sensitive pathways, mitochondrial integrity, and growth-factor–mediated protection, will be essential for optimizing NSC-based therapeutic strategies and identifying reliable biomarkers of NSC function in neurodegenerative disease.
